# Rapid and efficient gene delivery into the adult mouse brain via focal electroporation

**DOI:** 10.1038/srep29817

**Published:** 2016-07-19

**Authors:** Tadashi Nomura, Yusuke Nishimura, Hitoshi Gotoh, Katsuhiko Ono

**Affiliations:** 1 Developmental Neurobiology, Kyoto Prefectural University of Medicine, INAMORI Memorial Building, 1-5 Shimogamo-hangicho, Sakyoku, Kyoto, 606-0823, Japan; 2 Japan Science and Technology Agency, PRESTO, 4-1-8 Honcho, Kawaguchi, Saitama, 332-0012, Japan

## Abstract

*In vivo* gene delivery is required for studying the cellular and molecular mechanisms of various biological events. Virus-mediated gene transfer or generation of transgenic animals is widely used; however, these methods are time-consuming and expensive. Here we show an improved electroporation technique for acute gene delivery into the adult mouse brain. Using a syringe-based microelectrode, local DNA injection and the application of electric current can be performed simultaneously; this allows rapid and efficient gene transduction of adult non-neuronal cells. Combining this technique with various expression vectors that carry specific promoters resulted in targeted gene expression in astrocytic cells. Our results constitute a powerful strategy for the genetic manipulation of adult brains in a spatio-temporally controlled manner.

The transduction of adult tissue with exogenous genes is valuable for studying the genetic regulation of cell/tissue homeostasis. Currently, virus-mediated gene transfer is widely used for exogenous gene delivery, providing the robust expression of target genes in various tissues^1^,^2^,^3^. Additionally, conditional gene expression in transgenic animal has been accepted as a useful non-invasive strategy for genetic manipulation in postnatal and adult tissues^4^. However, these are time-consuming and expensive methods for achieving controlled gene expression. For example, the generation of high-titered virus particles for efficient gene delivery requires substantial labor and strict biosafety controls. Furthermore, the lack of cell type- or tissue type-specific promoters for restricted expression significantly hampers the spatio-temporal control of gene expression.

*In vivo* electroporation is a powerful technique that can be used to circumvent the problems associated with conventional strategies for gene manipulation^5^,^6^. The application of square electric pulses to tissues directly drives DNA into the inside of cells, allowing strong expression of exogenous genes. Currently, electroporation-based gene transfer has been broadly adopted as a convenient gene delivery method for a variety of cell types in developing and adult organs^6^. The electroporation of postnatal and adult brains has also been established in several groups^7^,^8^,^9^,^10^,^11^. However, high voltage electroporation of whole brains carries the risk of damaging tissues and inhibiting brain functions. Furthermore, targeting non-neuronal cells such as astrocytes has not been accomplished.

Here, we introduce a focal electroporation technique targeting the adult mouse brain. By localizing a syringe-based microelectrode directly into the lateral ventricle or brain parenchymal region, we achieved rapid and efficient gene transfer into non-neuronal cells such as ependymal cells and astrocytes with lower voltage than previous methods. The combination of this technique with specific promoters enabled cell type-specific gene expression in a spatio-temporally controlled manner. Our results provide a simple and powerful strategy for genetic manipulation in adult brains.

## Results

### Focal electroporation with a modified needle-type electrode enabled efficient transfection of the adult brain

**Figure 1:**
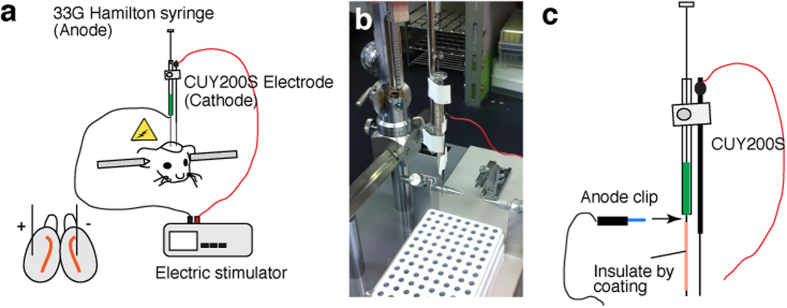
Focal electroporation of the adult SVZ with needle electrodes. (**a**) Schematic representation of focal electroporation. (**b,c**) The electrodes for focal electroporation. 33G Hamilton syringe and a needle type electrode (CUY-200S) are connected to the electric stimulator or electroporator. The metal needle of the syringe is insulated except for the tip and bottom. The anode clip is touched to the bottom of the gauge when electric pulses are applied to the animal.

**Figure 2:**
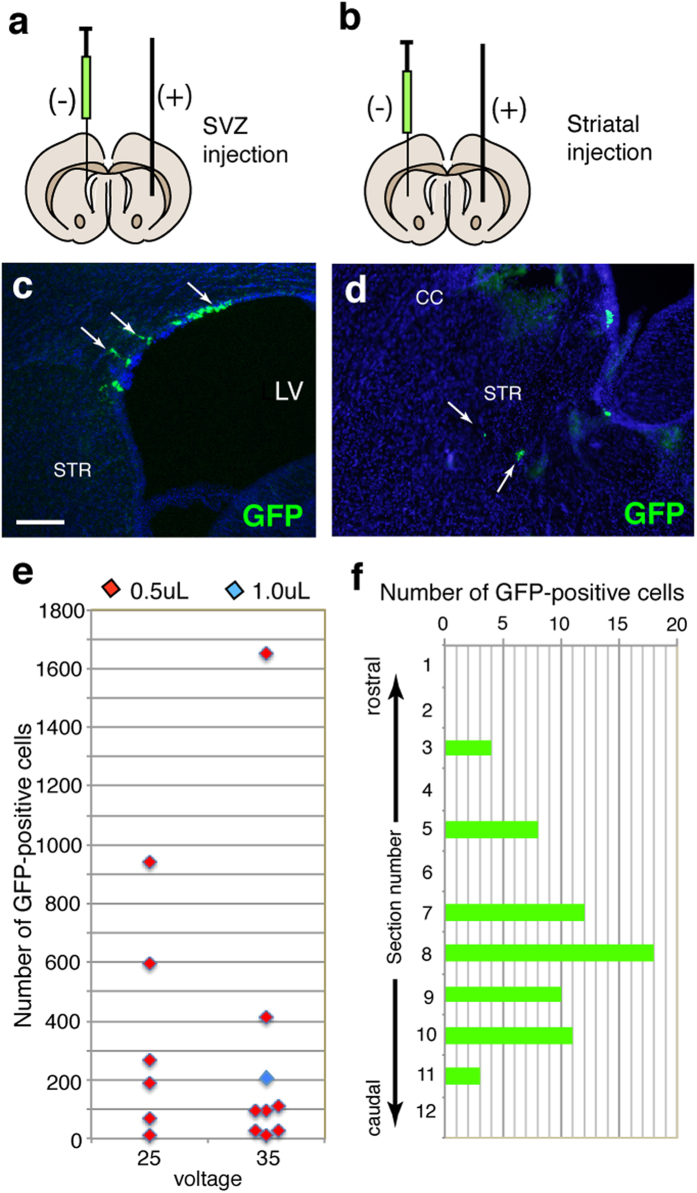
Distribution of electroporated cells. (**a,b**) Schematic representations of electroporation targeting the subventricular zone (SVZ; **a**) or striatum (**b**). (**c,d**) GFP-positive cells were distributed in the SVZ (**c**) or striatum (**d**). (**e**) The total number of GFP-positive cells via focal electroporation. (**f**) Distribution of GFP-positive cells in a representative animal. The number of GFP-positive cells in each section was obtained from the rostral to caudal axis of the brain. Scale bar: 100 μm in (**c**).

**Figure 3:**
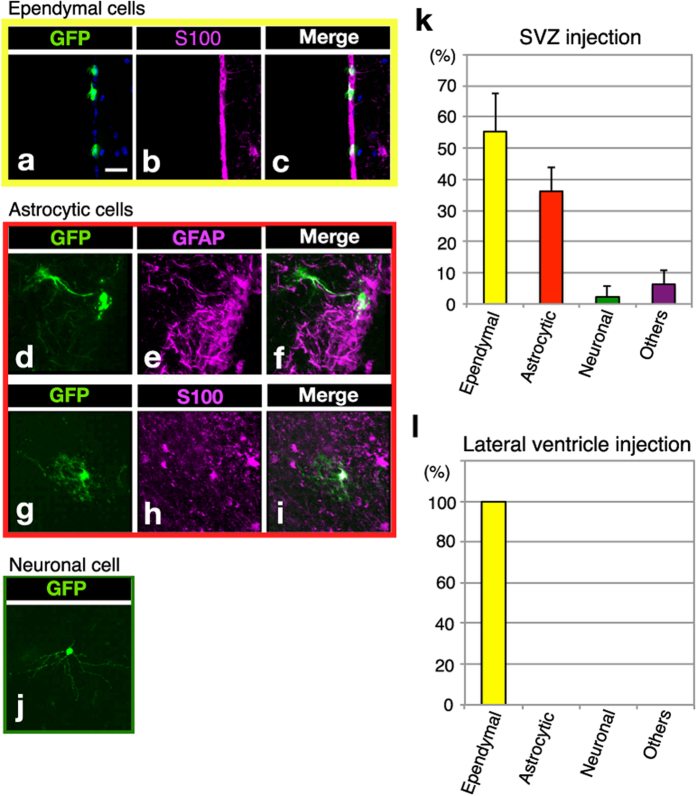
Characterization of GFP-positive cell types by immunohistochemistry. GFP-positive cells included S100ß-positive ependymal cells lining at the lateral ventricular wall (**a–c**), GFAP or S100ß-positive astrocytic cells (**d–i**) and neuronal cells (**j**). (**k,l**) The proportion of each cell type in the GFP-positive cells in animals with SVZ injection (**k**) or lateral ventricle injection (**l**). The GFP-positive cells were classified as ependymal cells, astrocytic cells, neuronal cells and others based on their morphology and marker expression. Scale bars: 25 μm (**a**).

The adult mammalian brain is composed of various cell types including neurons and non-neuronal cells. Although a previous electroporation method by intra-ventricular injection of DNA solution targeted adult brains, the majority of the transfected cells were ependymal cells that lining the surface of the lateral ventricle. In that case, the efficiency of transfection of non-ependymal cells was low (5.6% of total transfected cells)^11^,^12^. To increase the transfection efficiency of non-ependymal cells, it is necessary to inject DNA solution directly into brain parenchymal region. Furthermore, electroporation must be performed immediately after the injection, because the DNA solution cannot be sustained at the parenchymal region as it erupts from the injection track. A previous study indicated that a pair of needle-type electrodes (CUY200S) was useful for focal gene delivery into the parenchymal region of adult brains^8^. However, a pressure-controlled micro-pump was required to inject the DNA solution into the capillary of the anode, increasing the technical complexity of the experimental procedure. To circumvent this problem, we established a more convenient method for focal electroporation. A microliter syringe equipped with a metal gauge (701 SN: 33 gauge, Hamilton, Bonaduz, Switzerland) can be used to inject DNA directly into the tissue. Because the metal gauge of the syringe can be used as an electrode, we replaced the anode of the CUY200S with the Hamilton syringe and connected the bottom of the metal gauge to an electric stimulator (Fig. 1a–c). The surface of the metal gauge (with the exception of the bottom and the tip) was covered with nail polish for electricity insulation. A pair of electrodes was stereotactically inserted into the brain of an anesthetized adult mouse: an anode was directly targeted to the area close to the subventricular zone (SVZ) or striatal parenchyma, whereas a cathode was inserted into the contra-lateral side of the striatum (Fig. 2a,b). To minimize tissue damage, we slowly inserted electrodes (1 mm down/minute) and gently injected DNA solution (0.1 μl/minute). A vector expressing GFP under the control of a strong ubiquitous promoter (CAG vector contains CMV promoter and chicken ß actin enhancer) was used to monitor transfection efficiency. After 2 days of electroporation, we observed GFP-positive cells at the SVZ or striatal striatum, depending on the position of DNA injection (Fig. 2c,d). We tested two different voltages (25 V: n = 6 animals and 35 V: n = 9 animals), and confirmed that both voltages provided comparable results on the efficiency of transfection (GFP-positive cells: 344.66 ± 145.54 cells/animal in 25 V; 293.22 ± 173.93 cells/animal in 35 V; mean ± SEM) Fig. 2e. 0.5 μl of DNA solution (2.5~5 μg/μl) was sufficient to obtain a substantial number of GFP-expressing cells with minimum histological damage; increasing the amount of DNA solution enhanced tissue damages proximal to the injection tracks (Fig. 2e and data not shown). Although the number of GFP-labeled cells was higher proximal to the injection site, the GFP-positive cells were distributed in a broad area across the SVZ (over 300 μm in the rostral-caudal axis, Fig. 2f). To characterize the GFP-labeled cells, we performed immunostaining with several antibodies against cell type-specific markers (Fig. 3). Immunohistochemical analysis revealed that S100ß-positive ependymal cells lining the lateral ventricular wall were abundantly transfected (55.31 ± 7.14% of total GFP-positive cells in SVZ injection, and 100 ± 0% of total GFP-positive cells in lateral ventricle injection, Fig. 3a–c,k,l). In the case of SVZ injection, GFP expression was also detected by astrocytic cells, which were positive for glial fibrillary acidic protein (GFAP, Fig. 3d–f) or S100ß (Fig. 3g–i) and exhibited fibrous or protoplasmic morphologies. The transfection efficiency of astrocytes in the present method was markedly higher than that in the previous reports (36.12 ± 4.5% in the present method and less than 5% in the previous reports^11^,^12^; Fig. 3k). There were also some GFP-positive cells with long processes (a typical characteristic of striatal neurons) (2.29 ± 1.87%; Fig. 3j,k). These results indicate that the modified micro-electroporation enabled gene transfection of astrocytic cells in the adult SVZ more efficiently than previous methods.

### Validation of tissue damage after focal electroporation

**Figure 4:**
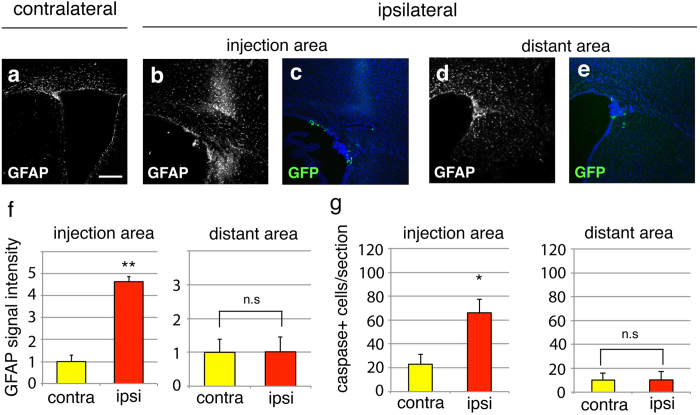
Validation of tissue damage and cell death after focal electroporation. (**a–f**) The expression of GFAP in the contra-lateral (**a**) and ipisi-lateral (**b–e**) brain regions of DNA injection. Reactive astrocytosis with elevated GFAP expression was evident at the injection area (**b,f**) but not at the distant area (**d,f**). (**g**) Quantification of active caspase9-positive cells in the electroporated brains. Scale bar: 100 μm in (**a**).

Next we assessed potential tissue damages due to micro-electroporation. After 2 days of electroporation, the expression level of GFAP was significantly increased in the ipsi-lateral side of the anode penetration track than on the contra-lateral side (Fig. 4a–c,f, n = 3 animals). Thus, reactive astrocytosis was induced on the ipsi-lateral side of the SVZ, particularly close to the injection track of the anode. However, the GFAP expression level in the brain was markedly reduced at areas distant from the electroporation site (>100 μm from the injection track). Of note, GFP-positive transfected cells were still detected at the subventricular areas that were distant from the injection track (Fig. 4d,e). A similar trend was also evident in the number of cells that were positive for activated caspase-3, a typical marker of apoptotic cells. Compared with the contra-lateral side, the number of caspase3-positive cells was significantly increased on the ipsi-lateral side of the anode penetration (66.12 ± 22.77 cells/section on the ipsi-lateral side and 23.0 ± 16.71 cells/section on the contralateral side; n = 4 animals, mean ± s.d., p = 0.027), whereas the number of these cells did not differ between the ipsi-lateral and contralateral sides at the distant areas (10.33 ± 7.09 cells/section on the ipsi-lateral side and 10.33 ± 5.53 cells/section on the contra-lateral side; n = 4 animals, p = 1.00, Fig. 4g). A slight gliosis was also detected on the contra-lateral side of the deep striatum near the cathode penetration (data not shown). However, we did not observe global damage in the contra-lateral brain. These data indicated that focal electroporation did not cause a widespread damage to the brain except for the area close to electrodes.

### Targeted gene expression using astrocyte-specific promoter

**Figure 5:**
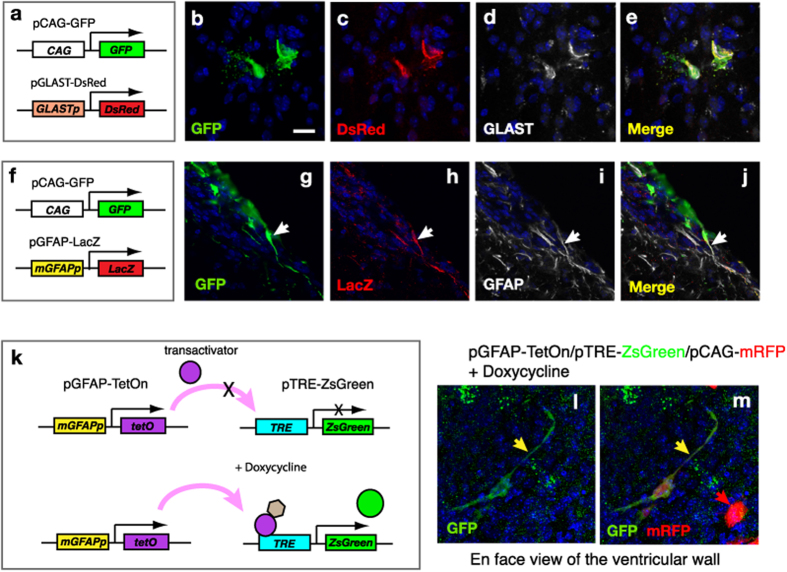
Electroporation of the expression vectors with astrocyte-specific promoter. (**a–e**) The electroporation of pGLAST-DsRed, in which DsRed was driven by rat GLAST promoter. GFP expression vector with CAG promoter was co-transfected with pGLAST-DsRed. After the focal electroporation, DsRed expression is detected in GLAST-immunoreactive cells. (**f–j**) The electroporation of pGFAP-LacZ in which ß-galactosidase was driven by mouse GFAP promoter. GFP expression vector with CAG promoter was co-transfected with pGFAP-LacZ. After the focal electroporation, LacZ expression was detected in GFAP-immunoreactive cells. (**k–m**) Doxycycline-dependent gene expression in astrocytic cells. The adult SVZ was co-transfected with pGFAP-TetON and the reporter plasmid (pTRE-ZsGreen) via focal electroporation (**k**). ZsGreen expression in an astrocytic cell (a yellow arrow) but not in an ependymal cell (a red arrow) after doxycycline administration (**l,m**). Scale bar: 25 μm in (**b**).

Although modified focal electroporation provided a higher efficiency of gene delivery into astrocytic cells, other cell types such as ependymal cells or neuronal cells were also targeted. To achieve astrocyte-specific gene transfection, we used cell type- specific promoter for astrocytic cells, such as astrocytic glutamate transporter (GLAST) or mouse GFAP. Vectors expressing dsRed or ß-galactosidase under the control of these promoters were delivered with pCAG-GFP vector into the SVZ (2.5 μg/μl; Fig. 5a,f). At 24 hours after electroporation, we observed restricted expression of reporters (pGLAST-dsRed, n = 2 animals; pGFAP-LacZ, n = 2 animals, Fig. 5b–e,g–j): this was in contrast to the broad expression of GFP driven by the ubiquitous promoter. Using specific antibody, we also confirmed that ds Red or ß-gal-expressing cells were positive for endogenous GLAST or GFAP by using specific antibodies: this validated the astrocyte-specific activation of these promoters after the electroporation (Fig. 5b–e,g–j).

To establish temporally controlled gene activation in astrocytic cell types, we utilized the Tet-On inducible gene expression system, which enables strong gene activation upon doxycycline administration. We constructed the expression vector to express tetracycline trans-activator (rtTA) under the control of GFAP promoter (pGFAP-TetON) and performed co-electroporation of the adult SVZ with a reporter vector that expressed a variant GFP under the control of a tetracycline-responsive element (pTRE-3G-ZsGreen) into the adult SVZ region (Fig. 5k). At 1 day after the focal electroporation, doxycycline was administered to the electroporated mice via drinking water for 2 days. At 3 days after electroporation, whole mount analyses of the lateral ventricular wall confirmed that ZsGreen was expressed in the restricted cell types with fibrous astrocyte-like morphology, whereas the RFP driven by the ubiquitous promoter was expressed in various cell types including ependymal cells (Fig. 5l,m). These results indicated that the combination of cell-specific promoters and an inducible expression system can be used for the spatio-temporally manipulation of gene expression via electroporation.

### Visualizing specific signaling activities in non-neuronal cells in the adult brain

**Figure 6:**
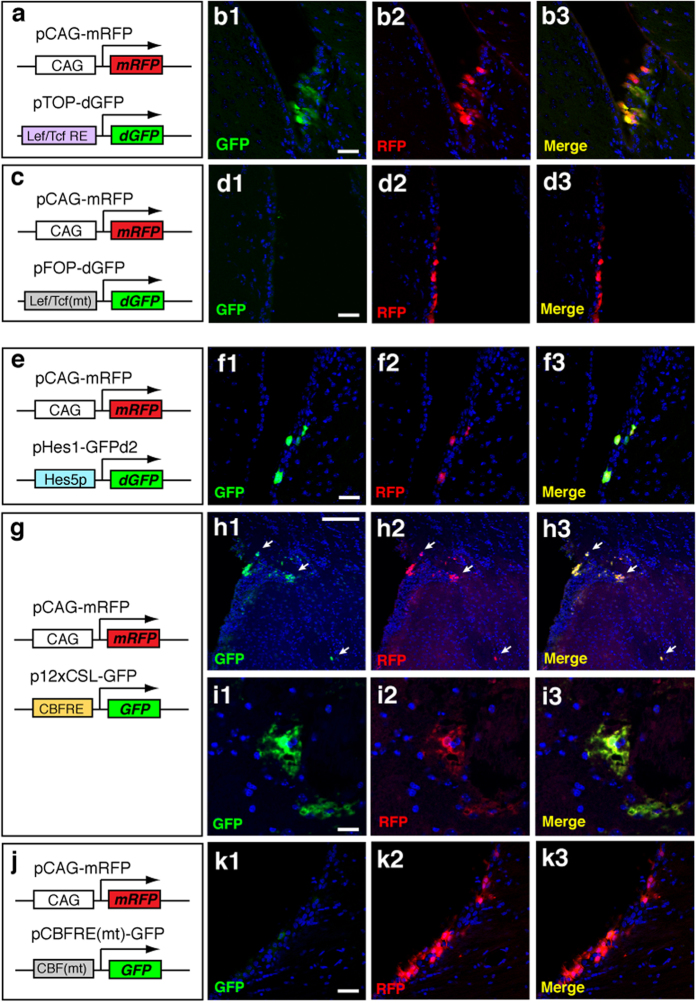
Monitoring intra-cellular signaling activity in the adult brain. (**a–b3**) Electroporation of a Wnt reporter vector into the lateral ventricle. RFP expression vector with CAG promoter was co-transfected with pTOP-dGFP that contains Lef/Tcf response elements (**a**). At 24 hours after electroporation, GFP expression was detected in ependymal cells by electroporation of pTOP-dGFP (**b1–b3**). Only a background GFP expression was detected by pFOP-dGFP that contains mutated Lef/Tcf response elements (**d1–d3**). (**e–f3**) Electroporation of pHes5-GFPd2, in which GFP tagged with de-stabilizing signal (**e**). After the focal electroporation, GFP expression was detected in ependymal cells (**f1–f3**). (**g–i3**) To monitor canonical Notch signaling activity, p12xCSL-GFP vector that expresses GFP under the control of CSL (CBF)-response elements, was electroporated to the SVZ (**g**). GFP expression was detected in the SVZ and striatum (arrowheads in **h1–h3**). An astrocytic cell that was positive for GFP (**i1–i3**). A background GFP expression was detected by electroporation of pCBFRE(mt)-GFP vector that contains mutated CBF response elements (**j–k3**). Scale bars: 25 μm in (**b1**), (**d1**), (**f1**), (**i1**) and (**k1**); 100 μm (**h1**).

Recent studies demonstrated that various intracellular signaling pathways play a pivotal role in the control of cellular characteristics in the adult brain^13^,^14^,^15^,^16^. Visualizing the activity of these signaling pathways in specific cell types is essential for the understating the regulation of cell fate maintenance and plasticity. Previous reports indicated that Wnt ligands-dependent signaling regulates cell proliferation and differentiation in the adult neurogenic region^15^,^17^,^18^; however, Wnt-dependent signaling activity in neurogenic niche cells, such as ependymal cells, has not been examined. To monitor Wnt signaling activity in ependymal cells, we electroporated a TCF/LEF reporter vector (TOP-dGFP), which expresses GFP in response to canonical Wnt signaling, into the lateral ventricle of the adult mouse brain (Fig. 6a). At 24 hours after electroporation of pTOP-dGFP, a strong GFP expression was observed in the ependymal layer of the electroporated brain (Fig. 6b1–b3). In contrast, a background level of GFP expression was detected by electroporation of a reporter vector containing mutated TCF/LEF response elements (pFOP-dGFP; Fig. 6c,d1–d3). These results implicate that canonical Wnt signaling is activated in ependymal cells. Next, we focused on Notch-dependent signaling pathway, which plays pivotal roles in the maintenance of ependymal cells and astrocytes in the adult brain^12^,^19^. To address Notch signaling activity, we used pHes1-GFPd2 vector and p12xCSL(CBF)-GFP vector, both of which direct GFP in response to canonical Notch signaling (Fig. 6e,g). By intra-ventricular injection of these vectors and subsequent electroporation, we confirmed a strong reporter expression in ependymal cells at 24 hours after electroporation (Fig. 6f1–f3). Furthermore, we detected GFP-positive protoplasmic astrocytic cells in the striatum by striatal injection of p12xCSL-GFP vector (Fig. 6i1–i3). In contrast, a background level of GFP expression was observed by electroporation of a reporter vector containing mutated CBF (CSL) binding sites [pCBFRE(mt)-GFP; Fig. 6j,k1–k3]. These data confirmed that Notch signaling is activated in ependymal cells and astrocytes, as previously reported^12^,^19^,^20^. Taken together, our electroporation method is applicable to detect the activity of various signaling pathways in specific cell types in the adult mouse brain.

## Discussion

*In vivo* electroporation targeting postnatal or adult brains has been reported by several groups^7^,^8^,^9^,^10^,^11^. Although previous methods provide rapid gene transfer into different types of brain cells, there were still technical problems such as the requirement for higher voltages and specific devices for accurate currency control. Here we present simple and efficient method for gene delivery into the adult brain by modifying micro-electroporation. Insulating the needle of the micro-syringe enables its use as a microelectrode, allowing the sequential injection of DNA solution and application of electric pulses. We succeeded in decreasing the voltage required for gene transfection compared with that required in previous reports; this modification substantially reduced potential damage to brain tissues, as well as postoperative animal mortality. Furthermore, our method resulted in efficient gene transfection of astroctytic cells; this results stands in contrast to previous reports that targeted neurons or ependymal cells^11^,^12^. This improvement could be due to the direct injection of DNA solution into the brain parenchymal regions, as well as the specific parameter settings of electroporation. Through the combination of cell-specific promoters and an inducible expression system, we established the controlled expression of exogenous genes in astrocytes. These results indicate several potential applications in studying the regulation of cellular characteristics in the adult brain. First, it is possible to perform rapid monitoring the activation and amplitude of specific signaling molecules through the use of promoter-reported constructions. Several signaling molecules, such as Notch, Wnt or Sonic hedgehog have been shown to be essential regulators for the proliferation and differentiation of neurons and glial cells in the adult brain; however, rapid *in vivo* systems monitoring these signaling cascades have not been established^14^,^21^,^22^. Second, using a transposase-mediated genetic recombination system, we can induce the stable expression of exogenous genes and permanent labeling of adult cell lineages without using specific viral vectors or transgenic strategies. Several transposase types, such as PiggyBac, Sleeping Beauty and Tol2 are currently available for *in vivo* transfection^23^,^24^,^25^, and a comparison of the recombination efficiency of these vectors could be relevant for the application of plasmid-based genetic labeling^26^.

An increase in GFAP expression, as well as the number of caspase-positive cells, was evident near the injection tracks; this implies the induction of reactive astrocytosis and apoptosis after injection of DNA injection and/or electroporaion. However, we confirmed that exogenous genes were delivered into broad areas around the SVZ, and we did not detect tissue damage or an injury reaction at areas distant from the needle tracks. Damage to local brain areas will be reduced by further methodological improvements, such as utilizing different types of electrodes or optimizing the electroporation parameters. Recent advances in plasmid-based technologies will enhance the utility of micro-electroporation and further elucidate the lineage relationships and molecular regulations of non-neuronal cells in the adult mammalian brain.

## Methods

### Animals

Adult C57BL/6J male mice (>8weeks) were purchased from Japan Charles River and maintained in our animal facility. All experimental procedures were approved by and performed in accordance with the Animal Research Committee of the Kyoto Prefectural University of Medicine.

### Plasmid vectors

pCAGGS-GFP^27^, pCAGGS-mRFP (a gift from Dr. Uchikawa), pDRIVE-mGFAP (Invivogen), GLASTp-DsRed2^28^ (addgene plasmid 17706), pLenti-TOP-dGFP^29^ (addgene plasmid 14715), pLenti-FOP-dGFP^29^ (addgene plasmid 14885), pHes1-GFPd2^30^ (addgene plasmid 14808), pCBFRE(mt)-EGFP^28^ (addgene plasmid 26870), pTRE3G-ZsGreen (Clontech), and p12xCSL-GFP^19^ were used for electroporation. To achieve tetracycline-specific reporter expression in astrocytic cells, Tet activator sequences were amplified from Tet-On3G vector (Clontech) via polymerase chain reaction (PCR) and fused with mouse GFAP promoter sequences isolated from pDRIVE-mGFAP. To induce tetracycline-dependent gene expression, doxycycline (20 mg/ml, Clontech) was administered into the electroporated mice via drinking water for 2 days.

### Electroporation

Mice were anesthetized with isoflurane (Escain, Mylan Pharmacy) by using an anesthesia unit (Univentor 410, BRC; gas flow was 250–300 mL/h, 3.0%). Under the anesthesia, electrodes were inserted into the brain of an anesthetized mouse with a stereotaxic frame (SR-6M, Narishige). Anode coordination for intra-ventricular injection was: 0 mm anterior/posterior, 1 mm right to the Bregma and 3 mm ventral to the dura. For targeting the SVZ: 0 mm anterior/posterior, 1.5 mm right to the Bregma and 3 mm ventral to the dura. For striatal injection: 0 mm anterior/posterior, 2 mm right to the Bregma and 3 mm ventral to the dura. The distance between the anode and cathode was 3 mm; the cathode was inserted into the contralateral side of the hemisphere with the same anterior/posterior and dorso/ventral relationship to the anode. After the injection of the DNA solution (2.5 μg/μL, total 0.5 or 1.0 μL), four times of square electric pulses (25 or 35 voltage, 50 ms for pulse-on period, 950 ms duration) were applied with the electric stimulator (SEN-3401, Nihon Koden) or pulse-generator (CUY edit II, BEX). After electroporation, the mice were recovered on a small animal warmer (BMT-100A, Bio Research Center).

### Immunohistochemistry

The anesthetized mice were perfused with with 4% paraformaldehyde (PFA) dissolved in phosphate buffered saline (PBS), and isolated brains were post-fixed with 4% PFA at 4°C for over night. After washing with PBS, the brains were cryoprotected with 30% sucrose solution, and embedded in Tissue-Tek. The frozen brains were sectioned at a thickness of 35 μm using a Cryostat (CM1900 and CM1850, Leica), and incubated with primary antibodies, including anti-GFP (rabbit polyclonal, Life Technology or rat monoclonal, Nakarai Tesque), anti-RFP (rabbit polyclonal, Abcam), anti-GLAST (goat polyclonal, Frontier Institute), anti-GFAP (mouse monoclonal, SIGMA), anti-ß-gal (rabbit polyclonal, MP Biomedicals), anti-activated caspase3 (rabbit polyclonal, Cell signaling), and anti-S100 (rabbit polyclonal, DAKO). After washing, the sections were incubated with secondary antibodies, including Alexa-Fluor 488, 594 or 633-conjugated anti-rabbit, anti-mouse, anti-rat and anti-goat antibodies (Life technologies). Nuclear staining was performed with Hoechst 33258. Sections were analyzed with either a fluorescent microscope (BX51, OLYMPUS) equipped with a cooled CCD camera system (DP71, OLYMPUS) or a laser confocal microscope (FV1000D, OLYMPUS).

### Image analyses

The expression of each marker was analyzed with a 40X objective lens of a laser confocal microscope, and image analyses were performed with ImageJ software. Statistical analyses were performed using Student’s two-tailed paired t-test.

## Additional Information

**How to cite this article**: Nomura, T. *et al*. Rapid and efficient gene delivery into the adult mouse brain via focal electroporation. *Sci. Rep.* **6**, 29817; doi: 10.1038/srep29817 (2016).
